# Analysis and mining of adverse events associated with vorapaxar: A FAERS database-based study

**DOI:** 10.1371/journal.pone.0340893

**Published:** 2026-02-13

**Authors:** Hui Zhang, Minghao Lin, Chaoqun Song, Zheng Nan, Dexi Zhao, Yujuan Fu

**Affiliations:** 1 Changchun University of Chinese Medicine, Changchun, China; 2 The Affiliated Hospital to Changchun University of Chinese Medicine, Changchun, China; 3 Acupuncture and Moxibustion Massage College, Jiangxi University of Chinese Medicine, Nanchang, Jiangxi, China; Celal Bayar University: Manisa Celal Bayar Universitesi, TÜRKIYE

## Abstract

**Objective:**

Vorapaxar is a platelet protease-activated receptor-1 antagonist that can inhibit thrombin- and thrombin receptor agonist peptide-induced platelet aggregation. This study aims to explore the potential medication risks of vorapaxar through data mining of its related adverse events, thereby providing a more rational and safe reference for clinical medication.

**Methods:**

In this study, adverse event reports related to Vorapaxar were retrieved and extracted from the FAERS database covering the 2004 Q1to 2024 Q4. The primary method employed was the reporting odds ratio (ROR) approach, which was used to detect risk signals associated with Vorapaxar.

**Results:**

A total of 185 adverse event reports were included in this study, among which male cases accounted for 52%, higher than the proportion of female cases. Most reports were submitted by consumers, and the majority of these reports originated from the United States. Screening of Vorapaxar identified 162 preferred terms (PTs), most of which were consistent with the adverse reaction information of Vorapaxar already published by the U.S. Food and Drug Administration (FDA).

**Conclusion:**

The adverse events involved 19 organ systems. Reports on vascular diseases, neurological diseases, and other conditions were numerous with strong signals, which were consistent with the drug instructions. Among them, vascular diseases had the highest risk of positive signals, including various hemorrhagic events and vascular structural/functional abnormalities. These findings suggest that clinical practice should be alert to adverse reactions in the vascular system, especially bleeding and severe vascular structural abnormalities.

## 1 Introduction

Atherosclerotic thrombotic diseases, as one of the leading causes of death and disability worldwide, have long been the focus of research in cardiovascular and related fields for their prevention and treatment [1]. Antiplatelet therapy serves as the core strategy to prevent recurrent thrombotic events in patients with such diseases [2]. Vorapaxar, a novel platelet protease-activated receptor-1 antagonist, with its unique mechanism of action—selectively inhibiting thrombin-induced platelet aggregation—provides a new therapeutic option for the secondary prevention of thrombotic diseases [3].

Based on the key evidence from the TRA 2°P-TIMI 50 trial, vorapaxar has been approved by FDA and the European Medicines Agency (EMA) for reducing the risk of thrombotic cardiovascular events in patients with a history of myocardial infarction or peripheral arterial disease [4]. Its additional benefits on top of standard dual antiplatelet therapy have attracted widespread attention. However, while the drug significantly reduces recurrent thrombotic events, it is accompanied by an increased risk of major bleeding, and this safety issue has always been a key factor requiring careful weighing in clinical application [5]. Subsequent VORA-PRATIC study further confirmed that vorapaxar can still effectively reduce platelet-driven thrombotic tendency even when used in combination with potent P2Y12 inhibitors, but at the same time, it again highlights the necessity of continuous monitoring of its adverse reactions [6].

Post-marketing safety monitoring of drugs is a crucial component in evaluating their risks in real-world applications. As one of the world’s largest public databases for adverse events, the U.S. FDA Adverse Event Reporting System (FAERS) collects drug adverse reaction reports from around the globe, providing important data support for identifying potential safety signals of drugs and detecting rare or delayed adverse reactions [7]. Although multiple clinical trials and reviews have explored the efficacy and safety of vorapaxar, large-sample, real-world data analyses based on the FAERS database remain relatively scarce [8]. Particularly in the field of encephalopathy, vorapaxar-related neurological adverse reactions (such as bleeding-related intracranial events) are closely related to clinical practice, and there is an urgent need to further clarify their occurrence characteristics, risk factors, and signal intensity through real-world data [9].

Therefore, this study aims to systematically analyze vorapaxar-related adverse event reports based on the FAERS database, with a focus on neurological adverse reactions, explore their potential safety signals, and provide real-world evidence support for rational clinical drug use, optimization of treatment regimens, and improvement of medication safety.

## 2 Methods

### 2.1 Data source and extraction

All our data comes from the FAERS Database (https://fis.fda.gov/extensions/FPD-QDE-FAERS/FPD-QDE-FAERS.html). FAERS database, with the extraction time range spanning from 2004 Q1 to 2024 Q4, covering the period from Vorapaxar’s first approval for marketing to the latest available report date. All data were from the public database, and no ethical approval was needed. The FAERS database includes fields such as patient demographic information (age, gender, etc.), medication information (drug name, route of administration, dosage, etc.), adverse event information (event name, occurrence time, outcome, etc.), and report sources (healthcare professionals, patients, pharmaceutical companies, etc.). Through the public data files of the FAERS database (including datasets such as DEMO, DRUG, REAC, and OUTC), all adverse event reports where Vorapaxar (generic name “Vorapaxar”) was identified as the primary suspect drug were extracted using a structured query method. The exclusion criteria were as follows: ① Reports that did not explicitly label Vorapaxar as the primary suspect drug; ② Reports with vague descriptions of adverse events or missing key information (e.g., no specific event name, no correlation with medication time); ③ Duplicate reports (identified through comparison of report numbers and core information).

### 2.2 Classification of adverse events and standardization of coding

The extracted adverse events were coded in a standardized manner with reference to the Medical Dictionary for Regulatory Activities (MedDRA, v26.1). Adverse events were hierarchically classified according to System Organ Classes (SOC) and PTs. Special focus was placed on the SOCs related to vascular disorders and nervous system disorders, and the corresponding PTs were extracted as the core adverse reactions of interest in the study (e.g., vascular operation, haematospermia, basal ganglia haemorrhage, etc.).

### 2.3 Data statistical analysis

To reduce the probability of false-positive signals, this study employed four methods—reporting odds ratio (ROR), proportional reporting ratio (PRR), Bayesian confidence propagation neural network (BCPNN), and empirical Bayes geometric mean (EBGM)—to assess the correlation between the drug and adverse events [10]. The larger the signal value of an adverse event, the stronger the signal of that adverse event, indicating a stronger correlation between Vorapaxar and the target adverse event. The calculation formulas and evaluation criteria of the four methods are detailed in Table 1. To improve accuracy, all PTs that meet the criteria in all four calculation methods are regarded as positive signals. Statistical analyses were performed using Python 3.9.10.

**Table 1:** Four major Methods used for signal detection.

Method	Formula	Threshold
ROR	ROR=(a × d)/(b × c)	a ≥ 3 ROR ≥ 2 95%CI (lower limit) > 1
	95%CI = e^ln(ROR)±1.96(1/a + 1/b + 1/c + 1/d)^0.5^	
PRR	PRR = a(c + d)/c/(a + b)	a ≥ 3 PRR ≥ 2 95%CI (lower limit) > 1
	χ^2^= [(ad-bc)^2](a + b + c + d)/ [(a + b)(c + d)(a + c)(b + d)]	
BCPNN	IC = log_2_a(a + b + c + d)/(a + c)/(a + b)	IC025 > 0
	95%CI = E(IC) ± 2V(IC)^0.5	
	r=(a + b + c + d)^2/(a + b + 1)/(a + c + 1)	
	E(IC)=log_2_a(a + b + c + d)^2/(a + b + c + d + r)/(a + b)/(a + c)	
	V(IC)=1/ln2(b + c + d + r-1)/(a + 1)/(a + b + c + d + r + 1)+(2 + b + c + 2d)/(a + b + 1)/(a + b + c + d + r + 3)	
	IC025 = E(IC)- 2V(IC)^0.5	
EBGM	EBGM = a(a + b + c + d)/(a + c)/(a + b)	EBGM05 > 2
	95%CI = e^ln(EBGM)±1.96(1/a + 1/b + 1/c + 1/d)^0.5^	
	EBGM05 = e^ln(EBGM)-1.96(1/a + 1/b + 1/c + 1/d)^0.5^	

Formula: a, number of reports containing both the suspect drug and the suspect adverse drug reaction; b, number of reports containing the suspect adverse drug reaction with other medications (except the drug of interest); c, number of reports containing the suspect drug with other adverse drug reactions (except the event of interest); d, number of reports containing other medications and other adverse drug reactions. ROR, reporting odds ratio; CI, confidence interval; N, the number of co-occurrences; PRR, proportional reporting ratio; χ^2^, chi-squared; BCPNN, Bayesian confidence propagation neural network; IC, information component; IC025, the lower limit of the 95% one-sided CI of the IC; EBGM, empirical Bayesian geometric mean; EBGM05, the lower 95% one-sided CI of EBGM.

## 3 Results

### 3.1 Descriptive analysis

**Fig 1:**
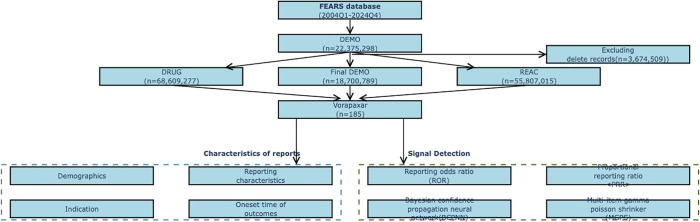
Reports of Vorapaxar.

A total of 185 reported cases associated with Vorapaxar were collected from the FAERS database, as shown in **Fig 1**. In terms of gender distribution, there were 96 male cases, 47 female cases, and another 42 cases with unknown gender. Among the 185 valid reports, age information was unavailable for 124 patients (67%). The following age distribution analysis is based on 61 samples with complete age data, showing that patients were predominantly aged 41–65 years and ≥65 years. The primary country of reporting was the United States. In terms of the reporters’ occupations, the majority were Consumers, accounting for 58.38% of the total reports. The most common outcome of adverse reactions was Hospitalization, accounting for 23.78%, followed by other serious reactions, accounting for 17.30%. The year 2015 saw the largest number of reported cases. Moreover, adverse reactions mainly occurred within 60 days after the administration of the drug, as shown in Table 2.

**Table 2:** Demographic characteristics of patients.

Characteristics	Case number/n	Case proportion/%
**Sex**		
Male	96	0.52
Femail	47	0.25
Unknown	42	0.23
**Age**		
<18	0	0
18-65	26	0.14
>65	35	0.19
Unknown	124	0.67
**Reporter**		
Consumer	108	0.58
Physician	32	0.17
Other health-professional	25	0.14
Pharmacist	19	0.10
Unknown	1	0.01
**Reporter Country**		
United States	181	0.98
Country not specified	4	0.02
**Outcome**		
Hospitalization	44	0.44
Other Serious	32	0.32
Death	12	0.12
Life-Threatening	6	0.06
Disability	5	0.05
Required Intervention to Prevent Permanent Impairment	1	0.01
**Time of taking the medication**		
<7	2	0.02
7-28	8	0.06
28-60	6	0.05
>60	9	0.07
Unknown	160	0.80

### 3.2 Disproportionality analysis

**Fig 2:**
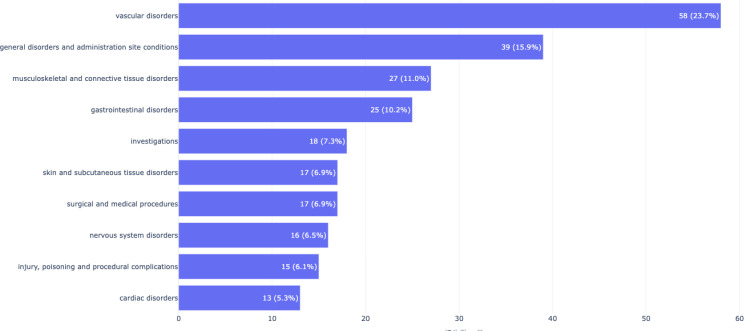
Top10 SOC of Vorapaxar.

Through the screening of Vorapaxar, a total of 162 positive risk signals of adverse events (AEs) were identified, covering 19 SOCs. A comparison with the current product instruction of Vorapaxar revealed that in terms of the number of reported AEs, 10 AEs were not listed, such as amyotrophic lateral sclerosis, back pain, surgery. Vorapaxar showed positive risk signals for AEs. According to the classification of SOCs, these AEs mainly affected 19 organ systems. Among them, vascular disorders ranked first, with a total of 29 positive risk signals of vascular – related AEs detected. According to the ROR ranking, Vorapaxar was mainly accumulated in the top ten SOCs, including vascular disorders, nervous system disorders, musculoskeletal and connective tissue disorders, surgical and medical procedures, investigations, cardiac disorders, skin and subcutaneous tissue disorders, injury, poisoning and procedural complications, general disorders and administration site conditions, and gastrointestinal disorders, as shown in **Fig 2**. The top ten PTs with relatively high signal intensity included: amyotrophic lateral sclerosis with ROR: 298.14, 95%CI (95.53,930.52), gastrointestinal haemorrhage with ROR: 34.27, 95%CI (20.04,58.6), epistaxiswith ROR: 30.96, 95%CI (16.95,56.52), dysstasia with ROR: 27.74, 95%CI (10.34,74.4), surgery with ROR: 15.52,95%CI (5.79,41.63), haemorrhage(site unknown) with ROR: 14.21,95%CI (6.71,30.06), rectal haemorrhage with ROR: 14.17, 95%CI (4.55,44.20), haematochezia with ROR: 11.51,95%CI (3.69,35.89), haemoglobin decreased with ROR: 9.88, 95%CI (4.08,23.91), drug dose omissionwith ROR: 9.13, 95%CI (4.53,18.44), as shown in **Table 3**.

**Table 3:** Positive signal and signal strength in Vorapaxar.

Preferred term (PT)	n	ROR (95% CI)	PRR (95% CI)	IC (IC025)	EBGM (EBGM05)
amyotrophic lateral sclerosis	3	298.14 (95.53,930.52)	295.19 (95.65,910.97)	8.2 (0.52)	294.72 (94.43)
gastrointestinal haemorrhage	14	34.27 (20.04,58.6)	32.73 (19.62,54.59)	5.03 (2.61)	32.72 (19.14)
epistaxis	11	30.96 (16.95,56.52)	29.86 (16.72,53.35)	4.9 (2.26)	29.86 (16.35)
dysstasia	4	27.74 (10.34,74.4)	27.38 (10.34,72.49)	4.78 (0.81)	27.4 (10.21)
surgery	4	15.52 (5.79,41.63)	15.33 (5.79,40.58)	3.94 (0.67)	15.33 (5.71)
haemorrhage(site unknown)	7	14.21 (6.71,30.06)	13.9 (6.68,28.91)	3.8 (1.36)	13.9 (6.57)
rectal haemorrhage	3	14.17 (4.55,44.20)	14.04 (4.55,43.30)	3.81 (0.26)	14.04 (4.50)
haematochezia	3	11.51 (3.69,35.89)	11.41 (3.70,35.17)	3.51 (0.20)	11.4 (3.66)
haemoglobin decreased	5	9.88 (4.08,23.91)	9.73 (4.08,23.21)	3.28 (0.78)	9.73 (4.02)
drug dose omission	8	9.13 (4.53,18.44)	8.92 (4.50,17.67)	3.16 (1.25)	8.9 2(4.42)

## 4 Discussion

### 4.1 Selection and validation of statistical methods

In the detection of drug adverse event signals, the rational selection of statistical methods directly affects the sensitivity and specificity of signal identification. This study adopted four methods: reporting odds ratio (ROR), proportional reporting ratio (PRR), Bayesian confidence propagation neural network (BCPNN), and empirical Bayes geometric mean (EBGM). The selection basis and verification logic are as follows [11].

As traditional frequency-based methods, ROR and PRR core principle is to calculate the association strength by comparing the proportion of adverse event reports between the target drug and other drugs in the database. Both methods are simple to calculate and have intuitive results, which are suitable for preliminary screening of potential signals, especially in quickly identifying high-frequency adverse events in large-sample data. Among them, ROR avoids small-sample bias by introducing a 95% confidence interval, while PRR focuses more on the direct quantification of proportional differences. The combination of the two can reduce the risk of missed detection by a single method.

BCPNN and EBGM are based on Bayesian theory, which correct data sparsity by introducing a prior distribution and are more suitable for detecting rare adverse events. BCPNN takes the information component (IC) as the core index and iteratively calculates the posterior probability through a neural network model, which can effectively reduce the interference of random fluctuations on signals. EBGM balances sensitivity and specificity through geometric mean and credible interval, and especially has better stability in identifying low-frequency events when the sample size is uneven.

This study included both traditional methods and Bayesian methods, aiming to meet the detection needs of adverse events with different frequencies and achieve comprehensive signal coverage. The selection of methodology in this study not only follows the consensus standards in the field of pharmacovigilance but also is optimized according to the clinical characteristics of Vorapaxar (such as prominent bleeding risk and the need to focus on neurological adverse reactions) [12]. For clinical practice in the department of encephalopathy, the sensitive detection of rare but serious events such as intracranial hemorrhage by BCPNN and EBGM can provide a more accurate basis for the evaluation of medication safety; while the analysis of high-frequency events by ROR and PRR is helpful to identify adverse reactions that are generally susceptible to the population and guide the adjustment of overall medication strategies.

The combined application of multiple methods combined with cross-validation can not only make full use of the value of real-world data in the FAERS database but also reduce bias through methodological complementarity, laying a methodological foundation for the clinical transformation of Vorapaxar adverse event signals.

### 4.2 Basic information on vorapaxar – related adverse event reports

In terms of gender distribution, the number of males reported cases (96 cases) was higher than that of female cases (47 cases), with another 42 cases having missing gender information. This proportional difference may be related to the characteristics of the clinical application population of Vorapaxar. It is known that Vorapaxar is approved for the secondary prevention in patients with myocardial infarction or peripheral arterial disease, and the incidence of such diseases is generally higher in men than in women, suggesting that the gender difference in reports may be partly due to the epidemiological characteristics of the underlying diseases. However, the proportion of missing gender information is relatively high (22.7%), so record bias during data collection needs to be considered. Future studies can further supplement demographic details by linking with electronic health records.

The age distribution shows that patients are mainly concentrated in the 41–65 years old and ≥65 years old age groups, which is consistent with the characteristics of Vorapaxar’s target population – atherosclerotic diseases are more common in middle-aged and elderly groups. It is worth noting that elderly patients (≥65 years old), as a high-risk group for bleeding, the proportion of their adverse event reports indicates that special attention should be paid to the medication safety of this group, especially the risk stratification management when they have multiple underlying diseases or use anticoagulant drugs in combination.

The main reporting country is the United States, which is closely related to the marketing time and market distribution of Vorapaxar (the drug was first approved in the United States in 2014) and reflects the regional tendency of the FAERS database. In the future, if adverse event databases from other countries or regions (such as the European Union’s EudraVigilance) are included, the consistency of Vorapaxar’s safety signals worldwide can be further verified.

Among the occupations of reporters, consumers account for as high as 58.38%, significantly higher than the proportion of reports from medical professionals. This phenomenon is not uncommon in drug safety monitoring directly facing patients. Although consumer reports can timely capture subjective discomfort symptoms (such as headache, dizziness, etc.), there may be problems such as non-standard event descriptions and deviations in causal relationship judgment, which need to be cross validated with medical records. In contrast, events reported by medical professionals usually contain more detailed clinical information (such as laboratory tests, imaging results), which is more valuable for judging the relevance of serious adverse events (such as intracranial hemorrhage).

Among the outcomes of adverse events, hospitalization (23.78%) and other serious reactions (17.30%) accounted for more than 40% in total, suggesting that Vorapaxar-related adverse events may have a significant impact on patients’ health. Combined with the drug’s mechanism of action, this result is consistent with the trend of increased bleeding risk observed in clinical trials, and special attention should be paid to the etiological composition related to bleeding in hospitalized cases (such as gastrointestinal bleeding, intracranial hemorrhage, etc.).

In terms of time distribution, the number of reported cases was the largest in 2015, which may be related to the “warning effect” in the early stage of the drug’s marketing – after a new drug is marketed, clinical attention is relatively high, and the reporting rate of adverse events usually has a short-term peak; while adverse reactions mainly occur within 60 days after medication, suggesting that monitoring should be strengthened in the early stage of treatment, especially during the dose adjustment or the initiation of combined medication.

In conclusion, the basic information of Vorapaxar-related adverse events not only reflects its application characteristics and known safety risks in the real world but also exposes potential biases in the data collection process (such as missing gender information, reporter heterogeneity) [13]. These findings provide a demographic and temporal reference framework for the subsequent in-depth analysis of risk factors for specific adverse events (especially neurological-related events) and also emphasize the importance of multi-source data integration in drug safety monitoring. The inherent limitations of real-world spontaneous reporting databases are the primary cause of this issue. Information in the FAERS database is derived from voluntary reporting by clinicians, patients, or pharmaceutical companies, and the lack of unified mandatory recording standards makes it prone to omissions of key clinical variables such as age and medication dosage. This missingness is not randomly distributed; instead, it may be associated with the attention paid by reporters and the accessibility of patients’ clinical data, thereby introducing selection bias. For instance, as an important confounding factor for bleeding risk (elderly patients typically have a higher bleeding risk), the substantial missingness of age data may hinder accurate adjustment for age-related effects on vorapaxar’s safety. Meanwhile, the lack of cumulative medication dosage data prevents exploration of the dose-response relationship, making it difficult to clarify differences in adverse event incidence across different dosage levels and weakening the guiding value of the study results for clinical individualized medication. Compared with including large-sample but highly missing data from the entire database, focusing on a subset with complete key variables (e.g., age, cumulative dosage) for core analysis can effectively reduce bias caused by missing values and improve the internal validity of the results. In subsequent analyses, we plan to prioritize selecting patients with clear age and complete cumulative medication dosage data to construct a core analysis cohort, ensuring the integrity of basic data and providing more reliable support for result interpretation.

### 4.3 Involvement of SOC by Vorapaxar – related AEs

This study found that Vorapaxar-related adverse event reports involve 19 organ systems in the MedDRA classification. The extensive involvement of these systems may be closely related to the pleiotropy of its mechanism of action. As a platelet protease-activated receptor-1 antagonist, Vorapaxar exerts its antithrombotic effect by inhibiting thrombin-induced platelet aggregation. Thrombin and its receptors are widely distributed in the body, not only participating in the coagulation process but also potentially involved in multiple physiological links such as vascular endothelial function and neural regulation, which provides a potential biological basis for the multisystem involvement of its adverse events.

Among various organ systems, vascular disorders, nervous system disorders, investigations, cardiac disorders, skin and subcutaneous tissue disorders, and general disorders and administration site conditions have a relatively large number of reports and high signal intensity [14]. This is basically consistent with the description in Vorapaxar’s instructions, further verifying the safety characteristics observed in clinical trials [15]. For example, the high incidence of vascular disease-related adverse events is directly related to the known bleeding risk of the drug, while the reports of nervous system diseases may be related to severe adverse reactions such as intracranial hemorrhage, which echoes the neurological safety issues suggested in the TRA 2°P-TIMI 50 trial and subsequent studies [16,17]. The high-frequency reports of abnormal investigation items may reflect the close monitoring of patients using Vorapaxar in clinical practice, such as regular detection of coagulation function, blood routine and other indicators, making it easier to capture relevant abnormal signals [18,19].

This study found that vascular disorders have the highest risk of positive signals and the most significant signal intensity [20]. This result further highlights the prominent position of vascular-related adverse events, especially bleeding events, in the process of antiplatelet therapy with Vorapaxar. Considering that the mechanism of action of Vorapaxar is to inhibit platelet aggregation, it may increase the risk of intravascular bleeding. Whether it is intracranial hemorrhage, gastrointestinal bleeding or bleeding in other parts, they all belong to the category of vascular diseases, which also explains why the signal of this system is the strongest [21]. This finding is consistent with the focus of clinical attention on the safety of Vorapaxar, suggesting that in clinical application, close monitoring of vascular-related adverse events in patients is needed, especially for patients with high-risk factors for bleeding.

This study also found several systems risks not mentioned in the instructions, including musculoskeletal and connective tissue disorders, surgical and medical procedures, injury, poisoning and procedural complications, and gastrointestinal disorders. These adverse event signals not covered in the instructions may originate from a wider population and longer medication time in the real world and can capture rare or delayed adverse reactions better than clinical trials. For example, the occurrence of gastrointestinal disease-related adverse events may be related to the impact of Vorapaxar on gastrointestinal mucosal blood vessels [22]. Although it was not focused on in clinical trials, it gradually appears in the large-sample application in the real world. For these newly discovered signals, further studies are needed to verify their causal relationship with Vorapaxar, and they also provide a reference for the update of instructions and clinical medication warnings [23].

### 4.4 Vorapaxar and other suspected PT signals

This study further clarified the specific preferred terms (PTs) of Vorapaxar-related vascular disorders, including amyotrophic lateral sclerosis, gastrointestinal haemorrhage, epistaxis, dysstasia, surgery, haemorrhage(site unknown), rectal haemorrhage, haematochezia, haemoglobin decreased, drug dose omission. The diversity and severity of these PTs not only confirm the conclusion mentioned earlier that the vascular system is a high-risk target for Vorapaxar-induced adverse events but also reveal the complexity of its vascular safety risks from the perspective of specific clinical phenotypes.

It is worth noting that there is a certain overlap in clinical symptoms between these vascular system PTs and the indications of Vorapaxar (such as gastrointestinal haemorrhage, epistaxis and haemorrhage disease). For example, peripheral vascular disorder is both the therapeutic target of the drug and may be reported as an adverse event. This “therapeutic contradiction” increases the difficulty of clinical judgment. In addition, abnormalities in relevant examination indicators (such as changes in coagulation function indicators) may result from the drug’s own effects, the progression of underlying diseases, or secondary infections, which further obscure the attribution boundary of adverse events. Therefore, when clinicians apply Vorapaxar, they need to comprehensively identify based on the patient’s medication history, underlying disease status, the temporal correlation of symptom occurrence, and auxiliary examination results, to avoid misjudging the natural course of the disease as a drug adverse reaction or missing the real drug-related adverse events.

### 4.5 Mechanism of vorapaxar

Vorapaxar, as the first clinically applied platelet protease-activated receptor-1 (PAR-1) antagonist, its unique mechanism of action is the core for understanding its clinical efficacy and characteristics of adverse events. PAR-1, as the main receptor for thrombin, plays a key role in platelet activation, regulation of vascular endothelial function, and inflammatory response [24]. Vorapaxar blocks the downstream G protein-mediated signaling pathway by selectively inhibiting the binding of PAR-1 to thrombin, thereby inhibiting platelet aggregation. This mechanism not only endows it with therapeutic value in antithrombosis but also lays a molecular foundation for the occurrence of its multisystem adverse events.

From the mechanism of hemorrhagic events, PAR-1 is in a core position in the cascade reaction of platelet activation, especially irreplaceable in the process of thrombin-induced platelet aggregation. The continuous inhibition of PAR-1 by Vorapaxar can significantly reduce the sensitivity of platelets to thrombin, resulting in the weakening of physiological hemostatic function. This effect is not limited to specific parts but systemic, which also explains the multiple-site hemorrhagic PTs observed in this study (such as basal ganglia hemorrhage, gastric hemorrhage, eye hemorrhage, etc.). It is worth noting that intracranial blood vessels are more sensitive to abnormal coagulation function. The basal ganglia region is rich in blood vessels with fragile structures. Against the background of decreased coagulation function caused by PAR-1 inhibition, coupled with the pressure of underlying diseases such as hypertension on the vascular wall, it is more likely to induce hemorrhage, which is highly consistent with the risk of intracranial hemorrhage that needs to be focused on in clinical practice of encephalopathy.

For adverse events related to vascular structural/functional abnormalities, their mechanisms may involve the extensive expression of PAR-1 in vascular endothelial cells. Under normal circumstances, after PAR-1 is activated by thrombin, it can promote vascular endothelial cells to release vascular protective factors such as nitric oxide (NO), maintaining vascular diastolic function and endothelial integrity. Long-term use of Vorapaxar may interfere with this balance: on the one hand, the inhibition of endothelial cell PAR-1 may weaken the self-repair ability of blood vessels and increase the risk of vascular wall damage (such as the formation of vascular pseudoaneurysms); on the other hand, the blocking of the thrombin-PAR-1 pathway may affect the proliferation and migration of vascular smooth muscle cells, thereby participating in the pathological process of arteriosclerosis or peripheral vascular diseases. In addition, the occurrence of aortic dissection may be related to the potential impact of the drug on vascular wall matrix metabolism. Although there is a lack of direct evidence at present, the role of PAR-1 in regulating the expression of matrix metalloproteinases (MMPs) suggests that its inhibition may damage the structural stability of the vascular wall.

Compared with traditional antiplatelet drugs (such as aspirin and P2Y12 inhibitors), the mechanism specificity of Vorapaxar is also reflected in its selective blocking of the “thrombin-PAR-1” pathway. Aspirin reduces the production of thromboxane A2 by inhibiting cyclooxygenase, and P2Y12 inhibitors block ADP-mediated platelet activation, while Vorapaxar targets the thrombin pathway, which is a more upstream activation signal. This makes its antiplatelet effect stronger, but it may also lead to more extensive impairment of hemostatic function. This difference may explain why the signal intensity of vascular system adverse events in this study is significantly higher than that of other systems, and the types of bleeding are more diverse.

However, there are still unclear issues at the mechanism level. For example, whether the occurrence of cerebral artery occlusion, as a thrombotic event, during Vorapaxar treatment is related to “drug resistance” or “thrombus rebound” caused by traditional antiplatelet drugs remains to be further studied. There is a hypothesis that PAR-1 inhibition may indirectly affect the properties of thrombus formation (such as thrombus stability) by altering platelet-leukocyte interactions, thereby inducing arterial occlusion under specific conditions. In addition, whether the occurrence of rare bleeding events such as haematospermia is related to the local PAR-1 distribution characteristics in the reproductive system or the accumulation of the drug in this part also needs to be verified at the molecular level.

### 4.6 Dual or triple therapy on bleeding risk

This study systematically analyzed the characteristics of vorapaxar-related adverse events based on large-sample data from the U.S. Food and Drug Administration (FDA) Adverse Event Reporting System (FAERS), providing real-world evidence support for the safe clinical use of this drug. However, in the process of interpreting the results, the limitations of the database itself and the impact of potential confounding factors need to be clearly clarified, among which the interference of combined antithrombotic therapy on bleeding risk is particularly worthy of in-depth discussion. In the field of antithrombotic therapy, dual or triple combination regimens (e.g., vorapaxar combined with aspirin and clopidogrel, or further combined with anticoagulants on this basis) are extremely common. This is especially true in patients with complex cardiovascular diseases such as acute coronary syndrome and atrial fibrillation complicated with coronary heart disease. Such combination regimens are aimed at enhancing antithrombotic efficacy, but they also significantly increase the risk of bleeding. Numerous clinical studies have confirmed that the combined use of antiplatelet drugs and anticoagulants can disrupt the balance between the coagulation and fibrinolysis systems by synergistically inhibiting coagulation factor activity or platelet function, leading to an exponential increase in the risk of serious adverse events such as gastrointestinal bleeding and intracranial hemorrhage.

In the adverse event analysis of this study, bleeding events were one of the main safety signals associated with vorapaxar. However, considering the characteristics of medication records in the FAERS database, we must be alert to the potential confounding effect of dual or triple antithrombotic therapy [25]. On the one hand, some reported bleeding events may not be caused solely by vorapaxar, but by the superposition of drug effects after combined medication. On the other hand, due to the lack of complete information in the database regarding patients’ baseline bleeding risk scores (e.g., HAS-BLED score, PRECISE-DAPT score) and comorbidities (e.g., gastrointestinal ulcer, renal insufficiency), we cannot accurately adjust for the interaction between these factors and combined therapy, which may lead to deviations in the assessment of vorapaxar-related bleeding risk. For instance, in the case of a patient receiving the combined therapy of vorapaxar + aspirin + rivaroxaban, it is difficult to clearly determine whether the resulting gastrointestinal bleeding is a specific adverse reaction of vorapaxar, or the combined effect of gastric mucosal damage caused by aspirin and the anticoagulant effect of rivaroxaban, relying solely on the existing information in the FAERS database.

Therefore, when interpreting the results of this study regarding vorapaxar-related bleeding risk, the potential impact of combined antithrombotic therapy must be fully considered. Although we have mentioned the limitations of the FAERS database in recording combined medication in the study limitations section, further strengthening the analysis of this confounding factor is of great significance. Firstly, it can provide more accurate medication references for clinicians, reminding them to strictly evaluate patients’ needs for combined antithrombotic therapy when prescribing vorapaxar, and avoid unnecessary polypharmacy. Secondly, it points out the direction for subsequent research. In the future, cohort studies based on electronic health records (EHR) can be constructed, combining complete medication history and baseline characteristic data to more accurately quantify the independent and interactive effects of vorapaxar and combined therapy on bleeding risk [26]. Thirdly, it provides ideas for pharmacovigilance work, suggesting that when using spontaneous reporting databases for signal mining, it is necessary to combine clinical practical medication scenarios and conduct stratified analysis of safety signals related to combined medication to improve the accuracy of signal identification.

Notably, despite the existence of the aforementioned confounding factors, this study still found that the incidence of vorapaxar-related bleeding events is higher in elderly patients and patients with renal insufficiency, which is consistent with the results of previous clinical studies. This suggests that individualized medication management for high-risk populations is crucial regardless of the presence of combined antithrombotic therapy. In clinical practice, clinicians should formulate precise treatment plans for patients based on balancing the benefits of thrombosis prevention and the risks of bleeding. Meanwhile, they should strengthen bleeding monitoring during medication, and establish a more rigorous follow-up mechanism especially for patients receiving multiple antithrombotic therapies. In summary, the analysis of this study based on the FAERS database provides important references for the real-world safety of vorapaxar, but the potential confounding effect of dual or triple antithrombotic therapy on bleeding risk deserves high attention. In the future, more rigorous prospective studies need to be designed, combining complete clinical information and medication details to further clarify the safety characteristics of vorapaxar, thereby providing more reliable evidence support for its rational clinical application.

## 5 Limitations

The FAERS database is a voluntary reporting system with reporting bias, which may lead to underestimation of certain adverse events (especially mild or non-serious ones), while serious or rare events may be over-reported due to high attention. Meanwhile, data such as demographic information, medication details, and comorbidities of some cases in the database are missing (e.g., 22.7% of cases in this study have unknown gender), which may affect the accuracy and stability of the analysis results [27]. In addition, the database lacks detailed information on the association between medication dosage, duration of medication and adverse events, making it difficult to conduct in-depth exploration of dose-effect relationships [28].

Furthermore, the mechanism discussion is mostly based on existing theories and hypotheses, lacking direct experimental evidence support. For example, the mechanism of adverse events related to vascular structural/functional abnormalities is only a speculative analysis, and the direct association between PAR-1 inhibition and changes in vascular wall matrix metabolism and smooth muscle cell function has not been verified through in vivo and in vitro experiments [29]. The mechanistic explanations for special adverse events such as cerebral artery occlusion and haematospermia also need to be confirmed by further molecular biology studies.

## 6 Conclusion

Adverse events related to Vorapaxar involve 19 organ systems in the MedDRA classification. Among them, systems such as vascular disorders and nervous system disorders have a relatively large number of reports and strong signals, which are basically consistent with the description in the instructions, further verifying the known safety risks of the drug. Vascular disease-related adverse events have the highest risk of positive signals, and their preferred terms include various hemorrhagic events and vascular structural/functional abnormalities. This suggests that clinicians need to remain highly vigilant about adverse reactions in the vascular system when using Vorapaxar, especially hemorrhagic events and severe vascular structural abnormalities.

The mechanism of selective inhibition of PAR-1 by Vorapaxar is the common basis for its efficacy and the occurrence of adverse events [30]. This mechanism exerts an antithrombotic effect by inhibiting platelet aggregation, but at the same time, it also weakens the physiological hemostatic function, leading to multi-site bleeding [29]. In addition, the impact on the functions of vascular endothelial cells and smooth muscle cells may be involved in the occurrence of adverse events related to vascular structural/functional abnormalities [31].

Given that there is a similarity between the indications of Vorapaxar and the symptoms of some adverse events, and the attribution of changes in relevant examination indicators is complex, clinicians need to make a comprehensive judgment based on the specific conditions of patients during application, carefully identify the nature of adverse events, so as to achieve individualized treatment and risk prevention and control [32,33].

This study provides real-world evidence support for the clinical safe use of Vorapaxar, but its limitations also suggest that more rigorously designed clinical studies and basic experiments need to be carried out in the future to further clarify the long-term safety characteristics of the drug, the dose-effect relationship, and the specific molecular mechanisms of adverse events, so as to provide more sufficient basis for optimizing clinical medication regimens and improving the safety of patients’ medication.
